# Algal Toxins Alter Copepod Feeding Behavior

**DOI:** 10.1371/journal.pone.0036845

**Published:** 2012-05-18

**Authors:** Jiarong Hong, Siddharth Talapatra, Joseph Katz, Patricia A. Tester, Rebecca J. Waggett, Allen R. Place

**Affiliations:** 1 Department of Mechanical Engineering, The Johns Hopkins University, Baltimore, Maryland, United States of America; 2 National Ocean Service, National Oceanic and Atmospheric Administration, Beaufort, North Carolina, United States of America; 3 Department of Biology, The University of Tampa, Tampa, Florida, United States of America; 4 Institute of Marine and Environmental Technology, University of Maryland Center for Environmental Science, Baltimore, Maryland, United States of America; Institute of Marine Research, Norway

## Abstract

Using digital holographic cinematography, we quantify and compare the feeding behavior of free-swimming copepods, Acartia tonsa, on nutritional prey (Storeatula major) to that occurring during exposure to toxic and non-toxic strains of Karenia brevis and Karlodinium veneficum. These two harmful algal species produce polyketide toxins with different modes of action and potency. We distinguish between two different beating modes of the copepod’s feeding appendages–a “sampling beating” that has short durations (<100 ms) and involves little fluid entrainment and a longer duration “grazing beating” that persists up to 1200 ms and generates feeding currents. The durations of both beating modes have log-normal distributions. Without prey, A. tonsa only samples the environment at low frequency. Upon introduction of non-toxic food, it increases its sampling time moderately and the grazing period substantially. On mono algal diets for either of the toxic dinoflagellates, sampling time fraction is high but the grazing is very limited. *A. tonsa* demonstrates aversion to both toxic algal species. In mixtures of *S. major* and the neurotoxin producing *K. brevis*, sampling and grazing diminish rapidly, presumably due to neurological effects of consuming brevetoxins while trying to feed on *S. major*. In contrast, on mixtures of cytotoxin producing *K. veneficum*, both behavioral modes persist, indicating that intake of karlotoxins does not immediately inhibit the copepod’s grazing behavior. These findings add critical insight into how these algal toxins may influence the copepod’s feeding behavior, and suggest how some harmful algal species may alter top-down control exerted by grazers like copepods.

## Introduction

Harmful algal blooms (HABs) are occurring with increasing frequency and magnitude across the globe, and have the potential to alter or disrupt ecosystem functions. In blooms of toxic or unpalatable phytoplankton, zooplankton grazing rates may decrease, thus negating top-down control and altering the transfer of energy to higher trophic levels [1]. *Karenia brevis* and *Karlodinium veneficum* are two common dinoflagellates that form HABs. *K. brevis* blooms are responsible for the infamous ‘red tides’ prominent in the Gulf of Mexico. It causes neurotoxic shellfish poisoning by producing brevetoxins, which are ladder polyethers that trigger numerous physiological symptoms in exposed organisms. *K. veneficum* is responsible for major fish kills in estuaries and brackish waters worldwide. It produces karlotoxins, which are pore-forming polyketides that cause cell lysis and death by increasing the membrane permeability. A recent study reported that karlotoxin was used by *K. veneficum* for capturing its *prey, Storeatula major* [2].

In the current research, we are interested in the interaction of calanoid copepods with toxic strains of *K. brevis* and *K. veneficum*. *Acartia tonsa* (Calanoida, Copepoda) is one of the most abundant zooplankters found within blooms of *K. brevis* [3], and is expected to be routinely exposed to background levels of *K. veneficum* in coastal waters of the southeastern USA [4]. Prior studies investigating copepod-dinoflagellate interactions have focused on using the armored dinoflagellate *Alexandrium*, which produces the neutotoxic saxitoxins. The role of this phycotoxin as a deterrent to zooplankton grazing was originally thought to be physiological impairment [5]–[7] and not particle rejection based on chemosensory response. Subsequently, [8], [9] showed, based on grazing statistics, that copepods could recognize and reject toxic cells prior to ingestion. Prey selectivity was indicated also by a number of behavior studies utilizing high-speed cinematography with 3D translation systems. [10] distinguished between raptorial and suspension feeding modes, and suggested that during raptorial feeding, the copepod detects ciliates from a long distance using their mechano or chemoreceptors. Investigations of turbulence effects on the prey detection and selection were performed successively [11]–[14]. Using tethered copepods when suspension feeding, [15] showed that the feeding current structure (e.g. high shear) could enhance the chemical signal detection. Recently, [16] observed an active prey selection of non-toxic prey over potentially toxic dinoflagellates.

A number of food removal and fecundity experiments focusing on interactions between *A. tonsa* and *K. brevis* (e.g. [17]–[19]) revealed reduced grazing for reasons that were not conclusively resolved. [17] found that *A. tonsa* avoided ingestion of *K. brevis* (unknown toxicity) and selectively ingested diatoms in a mixed bloom assemblage, causing starvation and reduced fecundity [18]. However, [19] argued that the decreasing grazing was more likely to result from nutritional inadequacy of *K. brevis*. Based on a grazing and survival experiment, [20] demonstrated that toxic *K. brevis* diet led to lower grazing rate, as well as higher mortality and egg production in comparison to diets of corresponding non-toxic strains. The only available grazing study of *A. tonsa* feeding on *K. veneficum* [4] showed a reduced grazing rate for toxic strains, but not to the same extent as those measured for *K. brevis*. The karlotoxins also had little effect on the copepod’s survivorship.

In this paper, we study the behavioral response of *A. tonsa* to varying diets of toxic and non-toxic isolates of *K. brevis* and *K. veneficum*, the same organisms used in [4], [20]. In all the current observations, predation occurs via suspension feeding, i.e. the copepod generates a feeding current to attract and capture prey [21]–[23] and not by raptorial feeding. We use digital holographic cinematography to simultaneously follow the 3D motion of numerous free-swimming copepods and prey cells. Statistical analysis of the duration of the copepods' feeding appendage beating enables us to distinguish between two beating modes in the suspension feeding behavior: “sampling beating” that typically occurs for a short duration and involves little fluid entrainment, and “grazing beating” that persists for longer periods and generates currents that entrain nearby prey cells. The fraction of time dedicated to sampling and grazing are strongly influenced by prey species present and the type of toxin they produce. These findings add critical understanding of how the mode of action of algal toxins may influence the copepods’ ability to exert top-down control, and help in terminating HABs [24].

## Materials and Methods

In our experiments, groups of twenty wild female *A*. *tonsa* were placed in a 30×30×100 mm^3^ transparent container, and allowed to swim freely. They were exposed to ten diets, as listed in Table 1, which included no-prey as a negative control, the cryptophyte *Storeatula major*, as a known good nutritional food source (positive control), as well as mono-algal and mixed, toxic and non-toxic strains of *K*. *brevis* and *K*. *veneficum. Supporting Information S1* summarizes the origin of the dinoflagellates used in the present study, as well as the properties of the toxins involved.

**Table 1:** The time fraction and mean duration of feeding appendage beating, hopping and escape reaction.

	no prey	*S. major*	*Karenia brevis*				*Karlodinium veneficum*				20 um polystyrene
			SP-1 (non-toxic)	2228 (toxic)	2228+*S.major*		1609 (non-toxic)	2064 (toxic)	2064+1609		
					1∶3	3∶1			1∶3	3∶1	
Total observation time (s)	437.09	398.28	484.04	575.36	416.9	366.44	457.31	391.97	711.2	658.89	54.6
Mean beating duration (ms)★	58±33	287±220	194±177	140±130	140±147	89±84	150±157	117±118	198±171	164±159	41±10
Cumulative beating fraction♦	0.022	0.320	0.262	0.134	0.078	0.034	0.177	0.143	0.220	0.158	0.008
Cumulative hopping fraction♦	0.0195	0.0201	0.0304	0.0221	0.0152	0.0191	0.0347	0.0292	0.023	0.0191	0.0108
Cumulative escape fraction♦	0.0036	0.0063	0.0134	0.0068	0.0123	0.0122	0.0127	0.0042	0.01	0.0112	0.005

★ The mean duration of a single beating event and its standard deviation.

♦ The total duration of a certain behavior divided by the observation time.

### Materials

*A. tonsa* were collected from Narragansett Bay, Rhode Island in July 2010 and in February 2009 from Pivers Island, Beaufort, North Carolina, USA by towing a 150 µm mesh, 0.5 m diameter plankton net, sorted by hand under a dissecting microscope and maintained in filtered seawater with sufficient food (*Rhodomonas salina*) until the day of experimentation. All the diet experiments were performed using the Narragansett Bay samples, except for the no-prey case, which involved the samples collected at Beaufort. These field locations are not privately-owned or protected in any way and our field studies did not involve endangered or protected species. No specific permits are required for collecting samples in these locations. All algal cultures were grown in standard media as described in [25], [26] (*S. major* and *K. veneficum*) and [20] (*R. salina* and *K. brevis*) to log phase and harvested for use in the behavioral studies.

### Experimental Setup and Procedures

**Figure 1:**
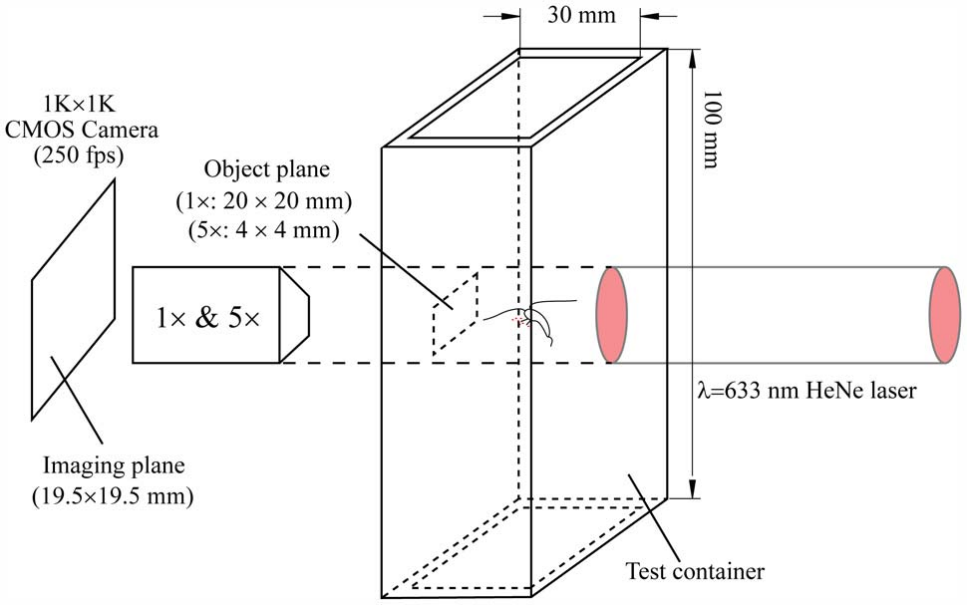
Experimental setup for in-line digital holographic cinematography.

Measurements were performed using inline, cinematic digital holography. This technique enabled us to simultaneously track numerous spatially-separated organisms, e.g. the copepods and prey cells, without loss of spatial resolution and over a long period in a 3D sample volume with a substantial depth. Figure 1 is a schematic of our experimental setup. Similar setups were used in a series of previous studies to examine the flow field around a feeding copepod [27] and the interaction of dinoflagellates with their prey [2], [28], [29]. The sample volume was illuminated by a collimated He-Ne laser beam (632.8 nm wavelength), and holograms at two different magnifications were acquired at 250 frames per second by a 1,024×1,024 pixels CMOS camera. Most of the *A. tonsa* behavioral data were acquired at a pixel resolution of 19.2 µm (1×). In addition, holograms recorded at a magnification of 5×, i.e. a resolution 4.1 µm/pixel were used to examine the interaction between *A*. *tonsa* and prey cells. Enhancement of the holograms before reconstruction included removal of time-invariant non-uniformities and equalization to correct for laser intensity variations. The holograms were numerically reconstructed using in-house developed software [2], [29] every 5 mm, which was sufficient for behavior classification, and every 20 µm for counting the dinoflagellate populations. At 1×, the reconstructed sample volume of 20×20×20 mm was located at the center of the transparent 30×30×100 mm^3^ container, i.e. the analysis did not include copepods (or prey) located within 5 mm from the walls.

Twenty fresh, well-fed, wild female *A. tonsa* were placed in the test container at the beginning of each diet experiment, which yielded a copepod concentration of 5 mls/copepod. This concentration, though high, was close to that the highest one used in [30], 6 mls/copepod, for which they showed little effect on copepods’ behavior. The dataset was obtained during three sequential recordings spaced 20 min apart, each consisting of 6144 holograms recorded during 24.6 s, which was the maximum data acquisition time allowed by the storage capacity of the high speed camera. The total duration of each diet experiment was about 40 min. Based on the reconstructed holographic movies, every single copepod was tracked continuously as long as it remained in the central sample volume, and we recorded the timing and duration of each behavior. With multiple simultaneous samples available, the cumulative (total) observation time for a single *A. tonsa* in each diet experiment ranged from 380 to 710 s (see Table 1). Using the reconstructed holograms, we directly measured the cell concentration in the sample volume at the start and the end of each diet experiment (see details in *Supporting Information S2*), and calculated corresponding clearance rates for 40 min duration. When referring to grazing rate, we rely on the longer duration grazing experiments [4], [20]. Although the intention was to set the start prey cell concentration at 1000 cells/ml for all diet cases, the measured initial concentrations varied. This concentration effect on the following data analysis was taken into account by using the cell concentration presented in Table 2, which was the mean of start and end concentration for each diet case.

**Table 2:** Sampling and grazing beating statistics.

	no prey	*S. major*	*Karenia brevis*				*Karlodinium veneficum*			
			SP-1 (non-toxic)	2228 (toxic)	2228+*S.major*		1609 (non-toxic)	2064 (toxic)	2064+1609	
					1∶3	3∶1			1∶3	3∶1
Cell concentration(cell/ml)	N/A	2039	1855	3373	2728	1844	1776	2414	2004	1723
Sampling beating fraction★	0.022	0.039	0.052	0.053	0.036	0.024	0.059	0.069	0.049	0.049
Grazing beating fraction★	0.000	0.281	0.209	0.082	0.042	0.010	0.118	0.073	0.174	0.109
Corr. sampling fraction♦	N/A	0.036	0.044	0.081	0.045	0.021	0.048	0.076	0.044	0.038
Corr. grazing fraction♦	N/A	0.302	0.248	0.053	0.034	0.011	0.146	0.066	0.190	0.139
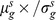 (ms)•	54×/1.88	75×/2.19	61×/1.95	65×/1.75	69×/1.98	52×/1.80	56×/2.07	61×/1.78	71×/1.94	69×/2.22
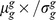 (ms)•	N/A	347×/1.66	235×/1.88	258×/1.51	342×/1.54	169×/4.97	275×/1.91	264×/1.48	254×/1.64	245×/1.51
rms fitting error – grazing◊	N/A	0.04	0.06	0.07	0.18	0.90	0.08	0.08	0.04	0.07

★ The total duration of sampling/grazing divided by the observation time.

♦ Values corrected for differences in prey cell concentration.

• For a normally-distributed variable, a domain consisting of ± standard deviation from the arithmetic mean contains 68.3% of the data. For a log-normally-distributed variable, 68.3% of the results fall within the range of the geometric mean ×/(multiply/divide) by the geometric standard deviation. Accordingly, 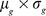 represents a confidence interval of 68.3% [38].

◊ errors resulting from log-normal fits.

### Behavior Categorization

**Figure 2:**
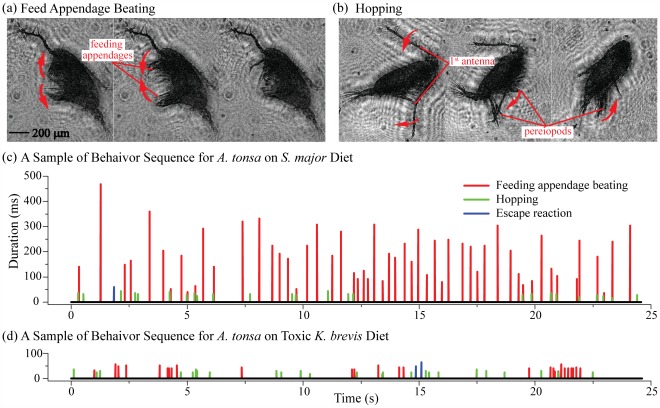
Sample holographic images and time series of *Acartia tonsa* behavior. Sample sequences of reconstructed images showing an *A. tonsa* performing: (a) feeding appendage beating, i.e. periodic movement of feeding appendages which include the 2^nd^ antennae, 1^st^ maxillae, 2^nd^ maxillae, mandibular palps and maxillipeds, shown at 4 millisecond (ms) interval, and (b) hopping involving a quick backward movement of both the 1^st^ antenna and pereiopods, shown at 8 ms interval. Escape reaction is characterized by repetitive motion of the pereiopods and retraction of the 1^st^ antennae, much like the last snapshot of the hopping sequence. (c) & (d) Samples of time series tracking the behaviors of a single *A. tonsa* when exposed to:(c) a mono-algal diet of *S. major*, and (d) a mono-algal diet of *K. brevis* 2228. For each event, the color and height of bars specifies the behavior and its duration respectively.

Based on our observations, and consistent with prior publications [31]–[33], *A*. *tonsa*’s behavior can be categorized as exhibiting free drifting, feeding appendage beating (Figure 2a, referred to as beating hereafter), hopping (Figure 2b), and escape reaction.

#### Feeding appendage beating

This behavior is characterized by periodic movement of feeding appendages which include the 2^nd^ antennae, 1^st^ maxillae, 2^nd^ maxillae, mandibular palps and maxillipeds. These feeding appendages operate in an integrated fashion to generate a flow field leading to prey entrainment and capture (Video S1). For each type of prey, we select 50 cells located initially within one copepod body length from the predator, in a conical volume extending from its mouthpart. We then measure the beating duration until the cell reaches the feeding appendages or the copepod stops appendage beating, whichever occurs first, along with the corresponding displacement of prey cells.

#### Hopping

This behavior is characterized by the quick backward movement of the 1^st^ antennae and pereiopods. These motions generate a strong thrust to enable the copepod to be translated quickly in a short distance or re-adjust its body orientation (Video S2).

#### Escape reaction

This behavior is characterized by the repetitive motion of the pereiopods and retraction of the 1^st^ antennae (Video S3).

A feeding appendage beating event is considered to terminate when the copepod retracts its feeding appendages and does not initiate further motion for a period of at least 12 ms, corresponding to three frames under our current recording speed. This criterion has been used to separate between sequential behavioral events throughout this study.

## Results

### Duration Statistics on *A. tonsa*’s Behavior

Two sample behavioral sequences showing the timing and duration of different behaviors demonstrate the frequency and variation possible during *A. tonsa* feeding on two extreme diet quality regimes. The first is *A. tonsa* grazing on an *S. major* CCMP 1868 (Figure 2c) and the second is *A. tonsa* grazing on a mono-algal diet of toxic *K. brevis* CCMP 2228 (Figure 2d). In a favorable food environment, the behavioral sequence contains frequent beatings for long durations (>200 ms), with hopping and escape reactions distributed almost uniformly throughout the period of observation. In contrast, when feeding on a toxic diet, the lengthy beatings are not evident, leaving primarily hopping and short-duration beatings. Combining the measurements of all tracked copepods, we use two parameters to quantify the frequency and total time fraction for different behaviors. The mean duration for beating, hopping and escape reaction are the average times for a single event. The cumulative fraction of each behavior refers to the total duration of this behavior of all the samples divided by the total observation time. For each behavior, in particular the beating, the duration of every behavioral event varies substantially. The duration histogram along with its mean and standard deviation are used to investigate the trends for the changing diets.

### Diet Induced Variations of *A. tonsa’s* Behavior

Some of the initial observations, consisting of cumulative time fractions of beating, hopping and escape reaction for all the experiments along with the corresponding mean duration are summarized in Table 1. Despite substantial standard deviations, the dependence of mean beating durations on diets is statistically significant (see *Supporting Information S3*). The longest mean beating duration and the highest cumulative beating fraction occur on the nutritional diet of *S. major*, consistent with the findings for suspension feeding [10]. On *K. brevis* diets, longer duration and higher beating fraction are observed with the non-toxic SP-1 strain than those with the toxic 2228 strain, in agreement with the trend of ingestion rates obtained during published food removal experiments [19], [20]. However, the results for the mixed-diets of *S. major* and *K. brevis* 2228 are puzzling, since the mean durations and time fractions do not fall between corresponding values of the mono-algal diets. Instead, mean durations and time fractions decrease to levels that are significantly lower than those occurring with the toxic *K. brevis* alone, and they decline further with increasing proportion of toxic *K. brevis*. Consistent with the food removal data from [4], the mean beating durations and cumulative fractions on the non-toxic *K. veneficum* CCMP 1609 diets are higher than those occurring in the presence of the toxic CCMP 2064 strain. However, the differences between the grazing responses on toxic and non-toxic *K. veneficum* are smaller compared to those associated with toxic and non-toxic *K. brevis* diets. Furthermore, in mixtures of toxic and non-toxic *K. veneficum, A. tonsa* does not significantly reduce its cumulative beating fractions or mean duration, in contrast to the *K. brevis* findings. Hopping and escape reaction behaviors (Table 1) show little correlation with food quality or toxicity, with the cumulative escape fraction being smaller than that of hopping for all diet cases. More details are provided in Supporting Information S3. In summary, the toxins and diets seem to affect only the A. tonsa’s feeding appendage beatings and not other behaviors in their repertoire. This statement refers to suspension feeding of *A. tonsa*, and does not contradict prior studies (e.g. [34]), which found that during raptorial feeding, the hopping and escape reactions were diet-dependent.

### Modes of Feeding Appendage Beating

**Figure 3:**
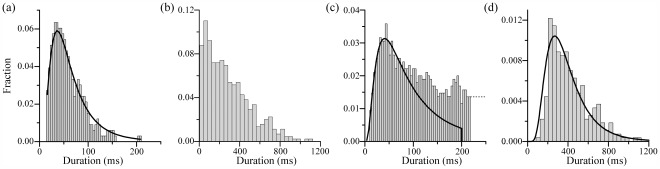
Decomposition of probability histograms of feeding appendage beating duration into sampling and grazing modes. Probability histograms of the duration of feeding appendage beating of *A. tonsa*: (a) sampling with no prey with 4 ms bins and (b) on an *S. major* diet with 40 ms bins. The solid line in (a) is the log-normal least square fit. (c) The 0–200 ms part of the histogram shown in (b) divided to 4 ms bins, along with the log-normal fit to the 0–100 ms range, which is terminated at 200 ms, and used as a model for sampling duration. (d) The conjectured grazing histogram on *S. major* diet obtained by subtracting the modeled sampling duration PDF shown in (c) from the data in (b). The solid line is the log-normal fit to the grazing duration histogram.

**Figure 4:**
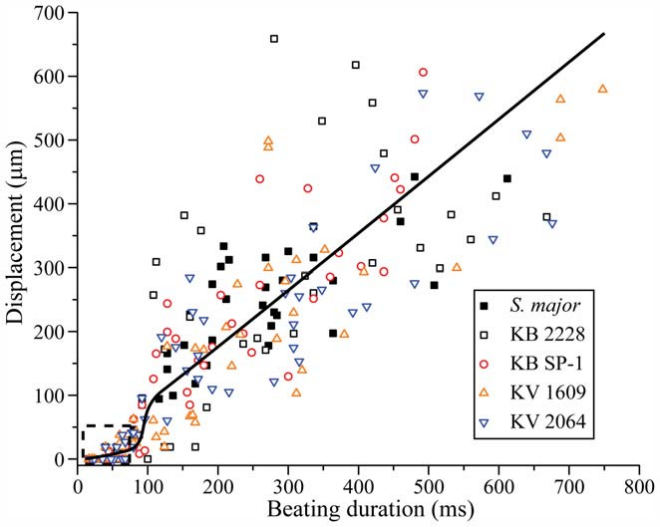
Relation between feeding appendage beating duration and prey cell displacement. Relation between feeding appendage beating duration and induced cell displacement in the vicinity of a single *A. tonsa*. The dashed box contains 82 data points. The solid line is a least square piece-wise linear fit of data points below and above 100 ms with smooth transition in between, showing the general trend of beating duration *vs.* cell displacement.

The duration histograms of beatings for *A. tonsa* on no prey (Figure 3a) and *S. major* (Figure 3b) diets, exhibit substantially different time spans, but the peaks of both are located in the vicinity of 44 ms. This observation holds for all the other diets, as demonstrated in the *Supporting Information S4* and *S5*. With little variation, a single 44 ms beating event consists of 3–4 periodic bouts of the feeding appendages. The relationship between beating duration and cell displacement (Figure 4) shows that, beating events that are shorter than 100 ms, do not cause displacements of more than 50 µm, i.e., one third of the characteristic length of the feeding appendage (∼150 µm). In particular, the durations that correspond to the peak of the histogram hardly induce any displacement. For longer periods, the displacements generally increase with beating duration. Furthermore, when *A. tonsa* is placed in a dense suspension of 20 µm diameter, nearly neutrally buoyant (specific gravity of 1.05) polystyrene particles, both the mean beating duration and cumulative beating fraction become very short (Table 1), and the corresponding particle displacement is very little. It is obvious from our holographic movies and consistent with published findings [35], that *A. tonsa* ignores the polystyrene particles as a food source. It should be noted that experiments involving the particles were performed at a different time, and are included here only to confirm that there is essentially no measurable feeding current for beatings with short duration.

When *A. tonsa* is placed in a no-prey environment, the beating duration is limited to the 0–200 ms range (Figure 3a), and 90% of the events are shorter than 100 ms. For such short beating durations, the copepods do not generate sufficient flow to capture/attract prey, as indicated by the above discussion. Imposing the energy minimization principle for biological organisms, there must be another reason for these beatings. We conjecture that they are used for screening and evaluating the environment by promoting the binding of odorant molecules with chemoreceptors concentrated along the copepod’s feeding appendages [36]. When prey are introduced (Figure 3b and *Supporting Information S4*), the beating histograms extend to much longer durations, ∼1100 ms in the *S. major* case, indicating that grazing involves beating durations exceeding the characteristic period of ∼100 ms. Thus, we separate the feeding appendage beating into two distinct modes: sampling and grazing. The short duration sampling mode persists in any environment, including cases of no-prey or polystyrene particles, and appears to have a “universal” probability density function (PDF) peak varying between 36 and 48 ms. In no prey case, the copepod might be responding to odorant molecules, such as dimethyl sulfide, that exist as chemical noise in the seawater [37]. When it senses prey, *A. tonsa* switches directly to a grazing mode that involves generation of a feeding current, whose duration is dependent on the local food environment.

In the analysis that follows, we use the no-prey distribution (Figure 3a) as a model for sampling behavior. Fitting a log-normal curve [38] to this duration histogram has a root mean squared (rms) error of less than 2%. We then infer that all the sampling durations have log-normal distribution with geometric mean values 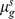 and standard deviation 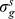 (superscript ‘s’ indicates sampling) that vary with the prey environment (see *Supporting Information S5* for all the duration histograms). Possible reasons for the log-normal distribution, which involve olfactory sensing by chemoreceptors, are present in the Discussion Section. To isolate the sampling part of each histogram, we assume that durations of less than 100 ms are used only for sampling, and that those longer than 200 ms are dedicated only to grazing. The magnitudes of 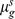 and 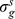 are estimated from a log-normal least square fit to the data in the 0–100 ms range, and the resulting sampling duration PDF is terminated at 200 ms (Figure 3c). This distribution is then subtracted from the total beating duration histogram. The remaining portion, shown in Figure 3d, is considered to be the grazing duration histogram. In view of its shape, we also perform a least-square fit to the grazing histogram with a log-normal distribution that results in a rms error of 4%.

**Figure 5:**
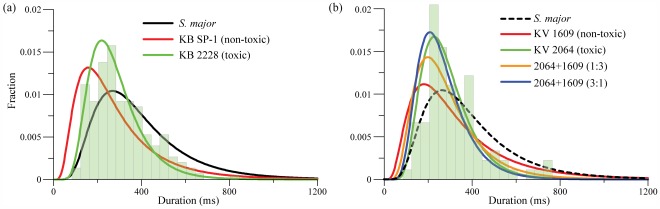
Probability density functions of grazing beating durations on different diets. Log-normal fitted curves of grazing beating duration for *A. tonsa* on diets of (a) *S. major*, *K. brevis* (KB) SP-1 and CCMP 2228, and (b) *K. veneficum* (KV) CCMP 1609, CCMP 2064 and mixed-algal diets of 2064 and 1609, with *S. major* as a reference. Bars: Grazing histograms for *A. tonsa* on *K. brevis* 2228 (a), and *K. veneficum* 2064 (b).

The same procedures and criteria are used to separate modes for all the diets. A complete set of histograms, curve fits, and their parameters are presented in *Supporting Information S5* and *S6*. Values of 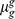 and 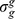 (superscript ‘g’ indicates grazing), as well as 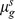 and 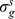 are also provided in Table 2. In the 0–100 ms range, the rms errors of the log-normal sampling fits vary between 0.2–2.6%, with negligible residues left for grazing (*Supporting Information S5*). Values of 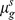 vary between 52 to 75 ms, and the corresponding most probable values, 38–48 ms, are very close to the measured ones (*Supporting Information S5*). Since there is no clear boundary between sampling and grazing on the duration histogram, we recognize that one might question the effect of specific choices used for defining the sampling PDF on the findings of this paper, especially the selected cutoff duration. We have tested another approach consisting of a single cutoff at 100 ms without log-normal fitting, i.e. beatings with duration less than 100 ms are considered as sampling, and ones with longer duration are considered as grazing. Different cutoff thresholds have also been tested for mode separation with details described in *Supporting Information S7*. As it demonstrates, selecting cutoff duration within a certain range (100–200 ms) only affects the specific values of grazing and sampling fraction, but not their general trends among diets. Fitting log-normal curves to grazing beating duration results in rms errors of less than 8% for all diets, except for the two cases involving mixtures of toxic *K. brevis* and *S. major.* There deviations are substantial (Table 2) and as a result, we do not use grazing curve-fits for these sets of data. As for the other seven grazing cases, 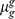 for the favorable *S. major* diet is distinctly higher than the rest, while the others values are close. The fitted curves of grazing durations, separated by dinoflagellate species, and with the *S. major* results as a reference, are presented in Figure 5. We opt to include the corresponding histograms for mono-toxic algae in each plot to demonstrate how well the log-normal fits represent the original distributions. In general, the PDFs broaden and their peak values decrease, indicating an increase in the characteristic grazing duration with increasing food “quality”.

### Dependence of Sampling and Grazing on Diet

The fractions of time dedicated to sampling and grazing, referred to as sampling and grazing fractions hereafter are provided in Table 2 for each diet case, and their sum equals the cumulative beating fraction in Table 1. Since the prey cell concentration is expected to affect the feeding activities [39], to compare trends of grazing and sampling fractions, it is essential to account for variations in cell concentrations (Table 2). For sampling, high prey concentration increases the encounter rates and binding rate of odorant molecules and chemoreceptors, presumably requiring a reduced screening time to evaluate the local environment. Conversely, the copepod is expected to increase its grazing rate with increasing concentration until reaching saturation levels [39]. Thus, to compensate for concentration effects, it is reasonable to assume as a first order approximation that the sampling time fraction is inversely proportional and that grazing time fraction is proportional to the prey concentration. Accordingly, the sampling and grazing fractions are linearly interpolated/extrapolated, using case-dependent slope, to 2195 cells/ml, the averaged value of all the present diet cases in which the cell concentrations are available (Table 2). Inherently, the no-prey results, which serve as a limiting reference case, are not corrected.

**Figure 6:**
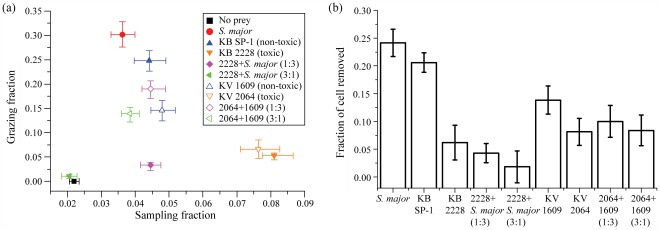
Sampling and grazing time fractions and fraction of removed prey cells on different diets. Sampling and grazing time fractions on different diets. The error bars represent 95% confidence interval based on a bootstrap method (*Supporting Information S9*). (b) The fraction of prey cells removed by *A. tonsa* during 40-min experiments. Error bars are based on the standard deviation of cell counts (Table 2).

Figure 6a illustrates how *A. tonsa* adjusts the time fraction allocated to each beating mode in response to varying diets based on the original (non-fitted) grazing data. The complementing Figure 6b compares the fraction of prey cells removed by copepods during the entire 40 min experiments, which is obtained by comparing the start and end cell counts.

Several trends become evident. First, when there is no food, the sampling time is low, and that of grazing is essentially zero. Switching to the favorable *S. major* diet, the sampling fraction more than doubles, and the grazing fraction peaks at 23%, with the results for the non-toxic *K. brevis* SP-1 being quite similar. Conversely, on mono-algal diets of both toxic dinoflagellate strains, the sampling fractions increase by five times from the no-prey level, while the grazing fractions remain low. *A. tonsa* does not attempt to entrain these prey cells, but continues to sample the water. In fact, the movies (Video S4 in *Supporting Information S8*) show that when a toxic *K. brevis* cell becomes inadvertently entangled with its feeding appendages, the copepod pushes the dinoflagellate away. Grazing suppression in the presence of toxic *K. brevis* is supported by previous food removal experiments [20] and the data in Figure 6b. However, we have occasionally seen *A. tonsa* ingesting toxic *K. brevis cell*. Consequently, the number of grazing events decreases with time during the 40 min experiment, presumably due to the toxicity of brevetoxin.

Second, the responses to mixed diets involving *K. brevis* are very different from those associated with *K. veneficum.* In mixtures of *S. major* with toxic *K. brevis*, both modes decrease in comparison to those of the toxic *K. brevis* alone, consistent with the fraction of prey cells removed (Figure 6b). In particular, for a dinoflagellate to *S. major* ratio of 3∶1, grazing almost disappears and sampling is reduced to the no-prey level. The cessation of beating is consistent with the poor long-term survival of *A. tonsa* in the presence of toxic *K. brevis* [20]. In contrast, on a mixed diet of *K*. *veneficum* cells, *A. tonsa* increases grazing and reduces sampling fraction to the level that is not significantly different from that of the non-toxic mono-algal diet. This trend agrees with the little difference in the fraction of *K*. *veneficum* cell removed among diets containing some non-toxic strain (Figure 6b).

## Discussion

### Grazing Suppression and Toxicity

A question raised from our results is why are grazing and sampling in mixed diets of *K. brevis* so low? To provide possible explanations, we note that evidence provided in several studies [9], [17] and our holographic movies (Video S5 in *Supporting Information S8*) show that *A. tonsa* can perform selective feeding. Thus, on a mixed diet *A. tonsa* might attempt to ingest the *S. major* cells, but in the process, it also ingests water containing brevetoxins, and occasionally toxic cells. Consequently, the copepod is more likely to be affected by the neurotoxic brevetoxins, in comparison to the mono-algal case. Indeed, in mixtures, *A. tonsa* becomes inactive after a short period (∼10 min), indicating a rapid neurological response, and does not exhibit a subsequent noticeable difference in behavior with time. An expectation that the intoxication level is related to the toxin concentration might explain the increased immobility with fraction of toxic *K. brevis* in the mixture. However, for the mixed diet of *K. veneficum*, although the longer grazing duration should increase the karlotoxin intake, evidently, it does not cause an appreciable effect on the beating characteristics during the present experiments. These observations are in agreement with the ability of *A. tonsa* to survive long periods on a diet of toxic *K. veneficum* [4], and the slow interactions of karlotoxins with membrane sterols [40].

In previous studies, the decrease in clearance rates of *A. tonsa* on toxic *K. brevis* has been attributed to behavioral avoidance [18], nutritional inadequacy [19] and physiological incapacitation [4], [20]. The presently observed short-term (40 min) behavior of a well-fed naive *A. tonsa* strongly suggests that ingesting the neurotoxic brevetoxin suppresses the feeding appendage beating behavior. On a diet of *K. brevis*, sampling is greatly enhanced, but on mixed diets, presumably once some of the toxins are ingested, both the sampling and grazing fractions decrease. Furthermore, the beating becomes feeble, i.e. *A. tonsa* lifts and retracts the feeding appendages only partially, but still maintains a beating frequency of about 60 Hz, the same as all the other cases. Although the low grazing and high sampling beating is also observed on a toxic *K. veneficum* diet, there is little difference between the copepod’s behavior and prey cell removal in non-toxic and mixed diet environments. Vigorous beating (fully-extended appendages) on mixed diets indicates that karlotoxin does not incapacitate the *A. tonsa*, at least in a short term. It is worth noting that the distinctly different impacts of brevetoxin and karlotoxin are consistent with mode of action of the toxins and trophic behavior of the dinoflagellates. The mixotrophic *K. veneficum* utilizes its toxins for immobilizing prey cells [2], but not as an effective means of inhibiting grazing by copepods. Conversely, as a predominantly autotrophic dinoflagellate, *K. brevis* captures prey infrequently [41], therefore the brevetoxin is employed largely as a grazing deterrent.

### Mode Separation and Log-normality

The mode separation is essential to explain copepods’ behavior on no-prey/polystyrene particle environment, in which cases the copepods predominantly perform short duration beatings that generate little food entrainment. One cannot explain this behavior without reasoning that the copepod is sampling the water, and since it detects little favorable food, it does not perform longer beatings that would entrain water and particles. In this way, the copepod utilizes less energy, and also avoids entrainment of toxic prey. On the mono-algal toxic diets, the sampling (but not the grazing) fractions are higher than all the other cases. A possible explanation is that the copepods might be sensing the presence of potential food (chemically or mechanically), and consequently, increase the sampling rate. However, since the feedback is unfavorable, they do not graze. This frequent sampling without entrainment might be a defensive strategy against intoxication. On *K. veneficum* diets, the mode separation enables us to show that the copepod does not favor the mono-algal diet of this species, and in fact, behaves similarly to that occurring on a mono-algal diet of *K. brevis*. If we did not separate the sampling and grazing beating modes, we would not be able to distinguish between behaviors on mono-algal toxic *K. veneficum* and mixed *K. veneficum* diets. In both cases, the total beating time fractions are close, but the time fractions of (short duration) sampling beatings are very different. On the mono-algal diet, the copepod performs a lot of sampling but little grazing. However, on the mixed diet, its sampling fraction decreases but the grazing fraction increases substantially.

The duration of both modes is well approximated using log-normal distributions, except for two cases, where intoxication disrupts the grazing behavior. The observed relationships between duration and diet are not sensitive to the separation approach (*Supporting Information S7*), and the trends in grazing beating generally agree with measured prey cell removal. These observations provide a simple approach for parameterizing the correlation between behavior and population dynamics in a pelagic planktonic ecosystem.

We can offer several explanations for why appendage beating behavior obeys a log-normal distribution. For example, a sampling beating event could be terminated when a threshold number of odorant molecules bind to chemoreceptors of *A. tonsa* to trigger a response. According to the Langmuir absorption isotherm [42], the receptor occupancy *X* has a hyperbolic dependence on the odorant concentration *C*, i.e. *X* = 1/(1+*k*_d_/*C*), where *k*_d_ is the disassociation constant for a particular ligand-receptor complex. Since 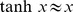 for 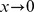 or 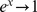, *X* = 1/(1+*k*_d_/*C*) = 0.5×[1–(*k*_d_/*C*–1)/(*k*_d_/*C*+1)]≈0.5×[1–0.51 ln(*k*_d_/*C*)] for *k*_d_/*C* is in the 0.1–10 range. This condition is usually satisfied for ligand-receptor binding [43]. The classical theory of receptor function [44] assumes proportionality of the drug response to receptor occupancy. Based on the above derivation, if *X* has a normal distribution, then, C, the concentration that would trigger a response, has a log-normal distribution. The chemo-sensing response of *A. tonsa* can be related to receptor occupancy in the same manner. When the threshold for chemo-sensing has a normal distribution, the concentration that would trigger this response has a log-normal distribution [43]. Equivalently, with increasing beating duration, the chemoreceptors bind to an increasing number of odorants, which in turn, reduces the number of unoccupied receptors, also resulting in hyperbolic dependence between beating duration and receptor occupancy, and a log-normal distribution of sampling duration. It is worth mentioning that the normality of chemo-sensing threshold can be understood from the following reasoning: Since the chemoreceptors of *A. tonsa* are spatially isolated [36], the amplitude of total odorant induced-current is the linear superposition of currents from individual channels [45]. According to the central limit theorem, when the amplitude of induced current in a single channel is random, the distribution of the total current amplitude and consequently the sensing response threshold, approach normality as the number of receptors increases. It should be noted that the normality of olfactory threshold has been demonstrated in human sensing [46], [47].

To explain the log-normal distribution of grazing duration, one could assume that grazing is terminated when a threshold number of prey cells are captured. We also assume that the filtering flux *Q* is constant in time, and that the capture zone has a shape of full cone with volume *V*, height *x*, cone angle *θ*, and apex located at the mouthpart of the copepod. Then, the induced feeding current is $Q\equals dV/dt\equals d\lpar \pi x^3 \tan ^2 \lpar \theta /2\rpar /3\rpar /dt$. Simple integration shows that the time required to entrain a fluid element located a distance *x* from the copepod is proportional to 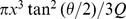. Based on [29], the nearest neighbor distance between dinoflagellates in a suspension has a normal distribution. Consequently, the grazing duration for capturing a prey cell is a cubic function of a normally-distributed variable, which can also be approximated by a log-normal distribution [43].

### Potential Ecological Impact

The food selectivity reported in earlier experiments and observed in our study is characteristically associated with coastal copepods, where both food and nutrients are more abundant [9], [17]. During the initiation of a *K. brevis* bloom, copepods are presumably exposed to a mixed assemblage of toxic dinoflagellates and other nutritional sources. As the concentration of toxic *K. brevis* increases to a near mono-specific bloom, selective feeding makes copepods more susceptible to neurotoxic brevetoxins, leading to a feeding inhibition that favors the bloom growth. Conversely, during the initiation of a toxic *K*. *veneficum* bloom, exposure to karlotoxins does not deter short-term grazing, and consequently copepods would be more effective in exerting top down control of the early bloom growth. However, as the *K*. *veneficum* bloom becomes mono-algal, the grazing slows down but does not cease. This diminishes, but does not eliminate the grazers’ impact on bloom development to the same extent. These findings are consistent with the differences in long-term copepod survival among these toxic dinoflagellates [4], and the macro-scale observation that *Karenia* blooms last from weeks to months while *Karlodinium* blooms are generally short lived [25], [48]. However, we remind the reader that the population dynamics that determines the formation and maintenance of harmful algal blooms is affected by a number of complicating factors, such as physical forcing [49] and long-term adaption of the grazers [50], [51].

## Supporting Information

Supporting Information S1 The properties of diets.
(DOC)

Supporting Information S2 Measurements of prey cell concentration and fraction of cell removed during short-term experiments.
(DOC)

Supporting Information S3 Statistical test for mean duration comparison of behaviors.
(DOC)

Supporting Information S4 Beating duration histograms for *A. tonsa* on *K. brevis* and *K. veneficum* diets.
(DOC)

Supporting Information S5 Sampling beating duration histograms for *A. tonsa* on *K. brevis* and *K. veneficum* diets.
(DOC)

Supporting Information S6 Grazing beating duration histograms for *A. tonsa* on *K. brevis* and *K. veneficum* diets.
(DOC)

Supporting Information S7 Sensitivity to mode separation criteria and procedure.
(DOC)

Supporting Information S8 Food rejection and selective feeding behavior.
(DOC)

Supporting Information S9 Bootstrap analysis of sampling and grazing beating fraction.
(DOC)

Video S1 A sample video of feeding appendage beating behavior.
(AVI)

Video S2 A sample video of hopping behavior.
(AVI)

Video S3 A sample video of escape reaction behavior.
(AVI)

Video S4 A toxic dinoflagellate becomes entangled with *A. tonsa*’s feeding appendages.
(AVI)

Video S5 *A. tonsa* performs selective feeding.
(AVI)
