# Supplementary material for: Algal Toxins Alter Copepod Feeding Behavior
Source: PLoS One. 2012 May 18;7(5):e36845. doi: 10.1371/journal.pone.0036845 (PMC3356345; doi:10.1371/journal.pone.0036845)
Supplement: Supporting Information S1 — The properties of diets. (DOC) [file pone.0036845.s001.doc]

**Supporting Information S1: The properties of diets**

**S1.1 Summary of all types of diets in the current study**

Table S1provides a summary of diets and their function in the current study.

**Table S1. Summary of the name, origin and purpose of all types of diets used in the present study**

| **Diets** | **Culture ID** | **Origin** | **Purpose** |
| --- | --- | --- | --- |
| *Storeatula major* | CCMP 1868 | Strain 'g' isolated from Chesapeake Bay in 1990 by Alan Lewitus(NOAA) | Good nutritional food |
| *Karenia brevis* | SP-1 | Port Aransas, Texas | Nontoxic |
| *Karenia brevis* | CCMP 2228 | New Pass, FL | Brevetoxic |
| *Karlodinium Veneficum* | CCMP 1609 | Choptank River, MD | Nontoxic |
| *Karlodinium Veneficum* | CCMP 2064 | Washington River, GA | Karlotoxic |
| *Rhodomonas salina*★ | CCMP 1319 | Milford, CT | Food supply |
| 20 μm polystyrene particles♦ | N/A | N/A | Check the feeding current during short duration beating |

Note: CCMP stands for Provasoli-Guillard National Center for Culture of Marine Phytoplankton.

★ This diet is used as a food supply for *Acartia tonsa*. No measurement was performed with this diet.

♦ This diet was used during experiments performed prior to the main grazing experiment of the present study. It is only employed to provide collateral evidence for some of our arguments.

**S1.2 Toxicity of diets**

Brevetoxins (PbTx) are lipid-soluble cyclic polyether compounds with a molecular weight of approximately 900 [1, 2]. There are >10 brevetoxin derivatives, which have all proven to be less toxic than the parent compounds of PbTx-1 and PbTx-2 [2, 3]. Brevetoxins bind with high affinity to receptor site 5 on the voltage-gated sodium channel (VGSC), and induce a channel-mediated Na^+^ ion influx [4, 5]. Neuro-excitation results from the nerve membrane depolarization and spontaneous firing. In some cases, only the nerve is depolarized, but both nerve and muscle depolarization have been noted [6]. The bevetoxin concentrations for the *Karenia brevis* used in the diet experiment were measured and provided in Table S2.

**Table S2. Brevetoxin measurement of non-toxic and toxic *Karenia brevis* strains**

|  | **PbTx-1 (pg / cell)** | **PbTx-2 (pg/cell)** | **PbTx-3 (pg/cell)** |
| --- | --- | --- | --- |
| *K. brevis* SP-1 | 0 | 0 | 0 |
| *K. brevis* 2228 | 0.8 ± 0.2 | 5.381 ± 0.8 | 0.178 ± 0.008 |

Karlotoxins are polar, lipid-like compounds that have hemolytic, cytotoxic and ichthyotoxic effects [7, 8]. Two karlotoxin variants, KmTx 1 and KmTx 2, have been identified, both acutely toxic to fish; however, KmTx 1 has been found only in strains of *Karlodinium veneficum* located in Chesapeake Bay waters while strains of *K. veneficum* in estuaries of the southeastern United States produce only KmTx 2 [8]. Karlotoxins increase the membrane permeability, leading to cell death through osmotic cell lysis [8]. In addition, they interact strongly with certain membrane sterols, in particular, cholesterol [9], which is the dominant sterol found in copepods, such as *Acartia tonsa* [10, 11]. The karlotoxin concentrations for the *K. veneficum* used in the diet experiment were measured and provided in Table S3. *K. veneficum* 1609 possesses very little amount of KmTx-2 compared with that from *K. veneficum* 2064. As a result, it is treated as a non-toxic *K. veneficum* strain in the present study.

**Table S3. Karlotoxin measurement of non-toxic and toxic *Karlodinium veneficum* strains**

|  | **KmTx-1 (pg / cell)** | **KmTx-2 (pg/cell)** |
| --- | --- | --- |
| *K. veneficum* 1609 | 0 | 0.002 ± 0.001 |
| *K. veneficum* 2064 | 0 | 2.1 ± 0.2 |

**References**

1. Shimizu Y, Chou HN, Bando H, Van Duyne G Clardy J (1986) Structure of brevetoxin A (GB-1 toxin), the most potent toxin in the Florida red tide organism *Gymnodinium breve* (*Ptychodiscus brevis*). Journal of the American Chemical Society 108: 514-515.

2. Baden DG, Bourdelais AJ, Jacocks H, Michelliza S Naar J (2005) Natural and derivative brevetoxins: historical background, multiplicity, and effects. Environmental health perspectives 113: 621-625.

3. Roszell LE, Schulman LS, Baden DG (1990) Toxin profiles are dependent on growth stages in cultured *Ptychodiscus brevis*. In: Graneli E, Sundstrom B, Elder L, Anderson DM, editors. Toxic Marine Phytoplankton. Elsevier, New York. pp. 403-406.

4. Poli MA, Mende TJ, Baden DG (1986) Brevetoxins, unique activators of voltage-sensitive sodium channels, bind to specific sites in rat brain synaptosomes. Molecular pharmacology 30: 129-135.

5. Trainer VL Baden DG (1999) High affinity binding of red tide neurotoxins to marine mammal brain. Aquatic toxicology 46: 139-148.

6. Watkins SM, Reich A, Fleming LE, Hammond R (2008) Neurotoxic shellfish poisoning. Marine drugs 6: 431-455.

7. Deeds JR, Terlizzi DE, Adolf JE, Stoecker DK (2002) Toxic activity from cultures of *Karlodinium micrum* (= *Gyrodinium galatheanum*)(Dinophyceae)- a dinoflagellate associated with fish mortalities in an estuarine aquaculture facility. Harmful Algae 1: 169-189.

8. Deeds JR, Reimschuessel R (2006) Histopathological effects in fish exposed to the toxins from *Karlodinium micrum*. Journal of aquatic animal health 18: 136-148.

9. Deeds JR Place AR (2006) Sterol-specific membrane interactions with the toxins from *Karlodinium micrum* (Dinophyceae) a strategy for self-protection? African Journal of Marine Science 28: 421-425.

10. Goad L (1981) Sterol biosynthesis and metabolism in marine invertebrates. Pure Appl. Chem 51: 837-852.

11. Ederington MC, McManus GB, Harvey HR (1995) Trophic transfer of fatty acids, sterols, and a triterpenoid alcohol between bacteria, a ciliate, and the copepod *Acartia tonsa*. Limnol Oceanogr 40: 860-867.
