# Supplementary material for: Algal Toxins Alter Copepod Feeding Behavior
Source: PLoS One. 2012 May 18;7(5):e36845. doi: 10.1371/journal.pone.0036845 (PMC3356345; doi:10.1371/journal.pone.0036845)
Supplement: Supporting Information S2 — Measurements of prey cell concentration and fraction of cell removed during short-term experiments. (DOC) [file pone.0036845.s002.doc]

**Supporting Information S2: Measurements of prey cell concentration and fraction of cell removed during short-term experiments**

The prey cell concentrations at the start and end of experiments for *A. tonsa* on each diet were measured by counting the number of cells in reconstructed hologram sets acquired during the first and last recording sequence of each experiment, that had a time gap ~ 40 min. Each set contained 6144 holograms recorded at 250 fps with an in-plane resolution of 19.2 µm/pixel, out of which 1 in 100 holograms was selected to be analyzed, yielding 61 holograms for each set. Each hologram was reconstructed in steps of 20 µm over the sample volume depth of 20 mm and the reconstructed images were intensity combined to obtain a single image with in-focus signatures of all particles in the sample volume. These combined images were then run through several image processing steps, such as intensity adjustment, histogram equalization, thresholding and 2D segmentation, which yielded a list of particles. This list was analyzed to remove particles with size and shape characteristics significantly different from the expected cell morphology. Since the total duration of each set was only 24.6 s, we did not attempt to distinguish between cell concentrations among the 61 holograms. The resulting mean and standard deviation of the concentrations are summarized in Table S4. For each diet case, the average cell concentration is defined as the average of the mean starting and ending cell concentration. This value is used for correcting/adjusting the sampling and grazing fraction in the main text. The fraction of cell removed is defined as the difference between the starting and ending concentrations divided by the starting value.

One might question the accuracy of measuring the concentration of *K. veneficum* cells, since its cell diameter is only about 70% of the pixel resolution. Noise becomes increasingly important in determining the accuracy of the measurement, as the maximum spatial frequency of the diffraction pattern to be recorded on a camera (dependent upon particle size) increases compared to the sampling frequency of the camera (pixel resolution). However, as discussed in [1], there is no sharp cutoff that determines what size can be resolved in the reconstructed images (nothing sacred about d being smaller or larger than the resolution). Intensity variation within a pixel is averaged over its area, so if the signal from a particle is strong enough (due to low-noise measurements, small sample volume, etc.), the intensity of this pixel can be distinguished from that of its neighbors. As demonstrated in Figure S1, a sample of reconstructed *K. veneficum* cell from our data can be clearly segmented from its background for cell counting, even though its size cannot be determined.

**Table S4**. **Cell concentration and fraction of cell removed for each diet experiment**

|  | Duration | Initial cell count | | End cell count | | Average cell count | Fraction of cell removed | |
| --- | --- | --- | --- | --- | --- | --- | --- | --- |
|  |  | Mean | Standard deviation | Mean | Standard deviation |  | Mean | Standard deviation |
|  | (min) | (cells/ml) | (cells/ml) | (cells/ml) | (cells/ml) | (cells/ml) | (%) | (%) |
| *S.major* | 40 | 2285 | 29 | 1792 | 21 | 2039 | 0.242 | 0.025 |
| *K. brevis*SP-1 | 41 | 2051 | 17 | 1659 | 16 | 1855 | 0.206 | 0.018 |
| *K. brevis* 2228 | 41 | 3483 | 61 | 3264 | 49 | 3373 | 0.063 | 0.031 |
| 2228+*S.major*(1:3) | 40 | 2791 | 25 | 2665 | 26 | 2728 | 0.044 | 0.017 |
| 2228+*S.major*(3:1) | 40 | 1862 | 31 | 1825 | 28 | 1844 | 0.019 | 0.029 |
| *K. veneficum*1609 | 39 | 1893 | 26 | 1659 | 16 | 1776 | 0.137 | 0.025 |
| *K. veneficum* 2064 | 41 | 2522 | 33 | 2306 | 31 | 2414 | 0.081 | 0.024 |
| 2064+1609(1:3) | 39 | 2100 | 28 | 1909 | 27 | 2004 | 0.99 | 0.028 |
| 2064+1609(3:1) | 40 | 1795 | 26 | 1651 | 22 | 1723 | 0.084 | 0.028 |

Note: For the cases with duration is not exactly equal to 40 min, the corresponding mean valued and standard deviation of fraction of cell removed are scaled proportionally to the values corresponding to 40 min duration.


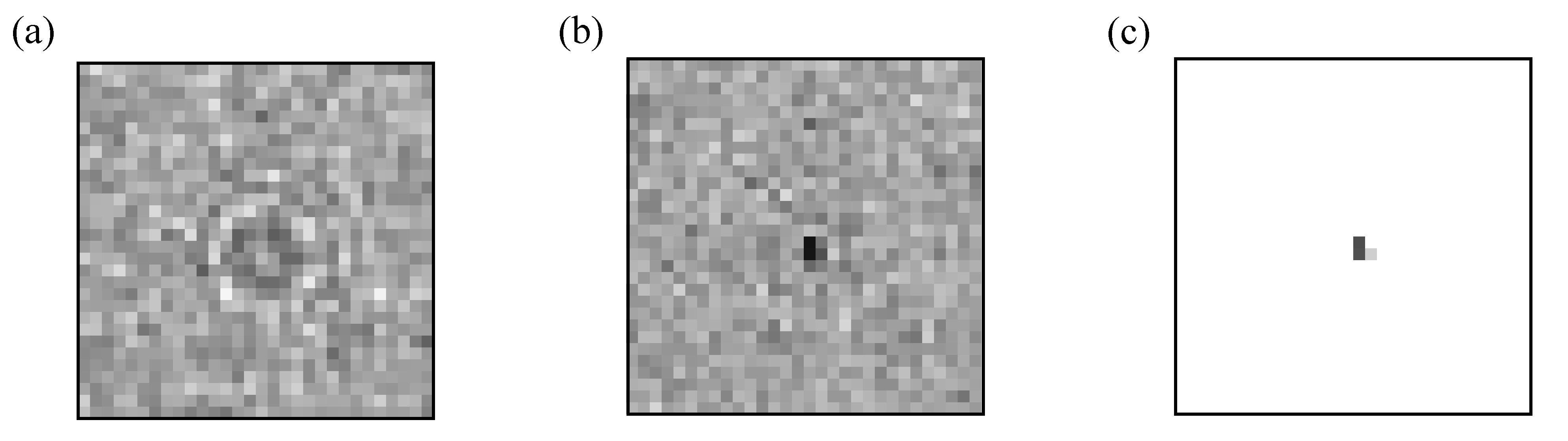


**Figure S1**. (a) The out-of-focus, and (b) the reconstructed in-focus image of a *K. veneficum* cell. (c) The segmented cell image from (b) used for cell counting.

**References**

1. Kelly DP, Hennelly BM, McElhinney C, Naughton TJ. A practical guide to digital holography and generalized sampling; 2008. Proc. SPIE. pp. 707215.
