# Supplementary material for: Algal Toxins Alter Copepod Feeding Behavior
Source: PLoS One. 2012 May 18;7(5):e36845. doi: 10.1371/journal.pone.0036845 (PMC3356345; doi:10.1371/journal.pone.0036845)
Supplement: Supporting Information S3 — Statistical test for mean duration comparison of behaviors. (DOC) [file pone.0036845.s003.doc]

**Supporting Information S3: Statistical test for mean duration comparison of behaviors**

The measured mean duration with standard deviation of feeding appendage beating, hopping and escape reaction for *A. tonsa* on each diet is presented in Table S5.

**Table S5** Summary of mean duration of feeding appendage beating, hopping and escape reaction

|  | no prey | *S. major* | *Karenia brevis* | | | | *Karlodinium veneficum* | | | | 20um polystyrene |
| --- | --- | --- | --- | --- | --- | --- | --- | --- | --- | --- | --- |
|  |  |  | SP-1 (non-toxic) | 2228 (toxic) | 2228+*S.major* | | 1609 (non-toxic) | 2064 (toxic) | 2064+1609 | |  |
|  |  |  |  |  | 1:3 | 3:1 |  |  | 1:3 | 3:1 |  |
| Mean feeding appendage beating duration (ms) | 58±33 | 287±220 | 194±177 | 140±130 | 140±147 | 89±84 | 150±157 | 117±118 | 198±171 | 164±159 | 41±10 |
| Mean hopping duration (ms) | 35±8 | 27±8 | 30±6 | 30±9 | 29±7 | 28±8.0 | 30±6 | 22±8 | 26±6 | 26±8 | 33±4 |
| Mean escape duration (ms) | 62±13 | 49±14 | 53±18 | 50±15 | 75±23 | 50±19 | 52±15 | 44±20 | 44±17 | 57±22 | 47±8 |

Since the beating duration histogram shows log-normality for *A*. *tonsa* on mono-algal diets. Kruskal-Wallis (non-parametric) one-way ANOVA with post-hoc Dunn's multiple comparison test, and t test on logarithmic-transformed data are applied to determine whether differences between their mean values are statistically significant (Table S6). P values less than 0.05 indicate that results are statistically significant. One-way ANOVA with Bonferroni multiple comparison test was also applied to the log-transformed data (nor present here). Since the ability to detect statistically significant difference with a non-parametric test is weaker than the parametric ANOVA on log-transformed data, so the fact that the non-parametric test is able to detect the differences is already very convincing. Although the log-transformed data do not strictly satisfy the assumptions for parametric ANOVA and t-test (i.e. normally distributed data which have homogeneity of variance), the results from these tests are still consistent with those from non-parametric tests.

**Table S6 Mean duration comparison of feeding appendage beating**

|  | Kruskal-Wallis one-way ANOVA with post-hoc test | | | t test on log-transformed data | | |
| --- | --- | --- | --- | --- | --- | --- |
|  | Difference in rank sum | p value | | t | df | p value |
| *S. major* vs *K. brevis* SP-1 | 347.7 | | <0.001 | 7.577 | 1098 | <0.001 |
| *S. major* vs *K. brevis* 2228 | 566.2 | | <0.001 | 12.43 | 997 | <0.001 |
| *K. brevis* SP-1 vs *K. brevis* 2228 | 218.5 | | <0.001 | 5.128 | 1207 | <0.001 |
| *S. major* vs *K. veneficum* 1609 | 582.8 | | <0.001 | 11.98 | 984 | <0.001 |
| *S. major* vs *K. veneficum* 2064 | 709.5 | | <0.001 | 15.03 | 919 | <0.001 |
| *K. veneficum* 1609 vs *K. veneficum* 2064 | 126.7 | | <0.001 | 2.438 | 1015 | 0.014 |

Since hopping and escape duration histogram approximate a Gaussian distribution, t-test is applied for comparing their mean durations (Table S7).

**Table S7** Mean duration comparison for hopping and escape reaction

|  | t test for mean duration of hopping | | | t test for mean duration of escape reaction | | |
| --- | --- | --- | --- | --- | --- | --- |
|  | t | df | p value | t | df | p value |
| *S. major* vs *K. brevis* SP-1 | 7.27 | 781 | <0.001 | 1.17 | 172 | 0.244 |
| *S. major* vs *K. brevis* 2228 | 5.87 | 715 | <0.001 | 0.2042 | 128 | 0.839 |
| *K. brevis* SP-1 vs *K. brevis* 2228 | 0.1244 | 898 | 0.901 | 1.063 | 161 | 0.289 |
| *S. major* vs *K. veneficum* 1609 | 0.4957 | 1005 | 0.621 | 1.503 | 86 | 0.136 |
| *S. major* vs *K. veneficum* 2064 | 8.634 | 818 | <0.001 | 1.138 | 200 | 0.257 |
| *K. veneficum* 1609 vs *K. veneficum* 2064 | 19.76 | 1042 | <0.001 | 2.616 | 147 | 0.010 |
