# Supplementary material for: Algal Toxins Alter Copepod Feeding Behavior
Source: PLoS One. 2012 May 18;7(5):e36845. doi: 10.1371/journal.pone.0036845 (PMC3356345; doi:10.1371/journal.pone.0036845)
Supplement: Supporting Information S4 — Beating duration histograms for A. tonsa on K. brevis and K. veneficum diets. (DOC) [file pone.0036845.s004.doc]

**Supporting Information S4: Beating duration histograms for *A. tonsa* on *K. brevis* and *K. veneficum* diets.**

The entire set of feeding appendage beating duration histograms for *A. tonsa* on all the mono-algal and mixed diets of *K. brevis* and *K. veneficum* are presented in Figure S2.


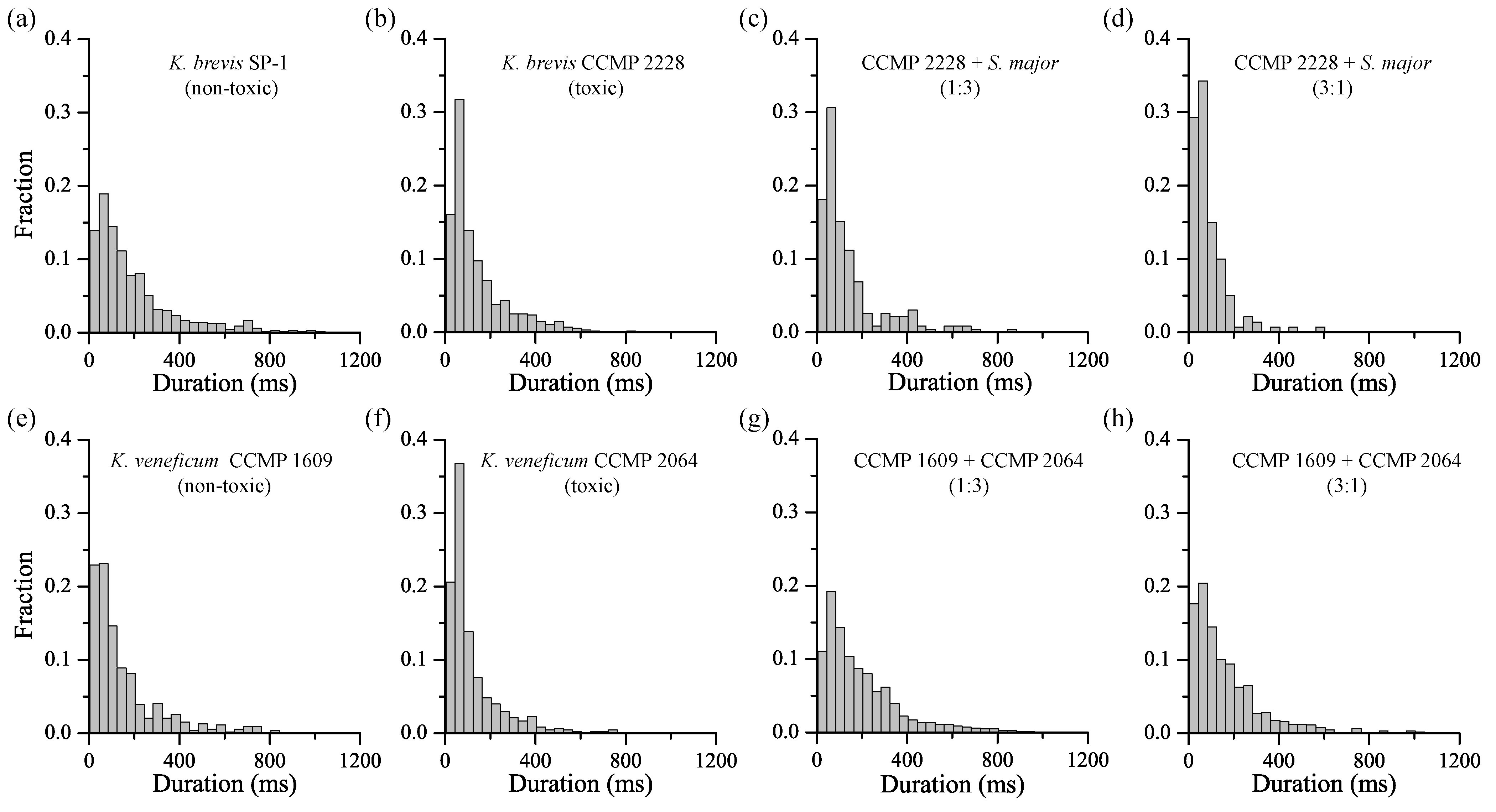


**Figure S2**. Beating duration histograms for *A. tonsa* on mono-algal and mixed diets of *K. brevis* and *K. veneficum*. Top and bottom row correspond to *K. brevis* and *K. veneficum* type of diets, respectively. The sampling bin size is 40 ms.
