# Supplementary material for: Algal Toxins Alter Copepod Feeding Behavior
Source: PLoS One. 2012 May 18;7(5):e36845. doi: 10.1371/journal.pone.0036845 (PMC3356345; doi:10.1371/journal.pone.0036845)
Supplement: Supporting Information S5 — Sampling beating duration histograms for A. tonsa on K. brevis and K. veneficum diets. (DOC) [file pone.0036845.s005.doc]

**Supporting Information S5: Sampling beating duration histograms for *A. tonsa* on *K. brevis* and *K. veneficum* diets**

Figure S3 shows parts of the feeding appendage beating duration histograms for *A. tonsa* on *K. brevis* and *K. veneficum* diets, focusing on the 0−200 ms range. As described in detail in the main text, these histograms are least-square fitted with log-normal curves,

$$f(x)=\frac{1}{x\cdot\sigma\sqrt{2\pi}}\exp\left(-\frac{1}{2\sigma^{2}}(\log(x)-\mu)^{2}\right)$$

using the data in the 0-100 ms time range. These fitted curves are terminated at 200 ms, and the truncated distribution is used for obtaining the values of the geometric mean, $\mu_{g}^{s}$, and standard deviation, $\sigma_{g}^{s}$. The properties of the fitted curves along with the duration corresponding to the peak of the original histograms are provided in Table S8. Note that for a log-normal distribution, the geometric parameters are and where *μ* and *σ* are arithmetic counterparts.


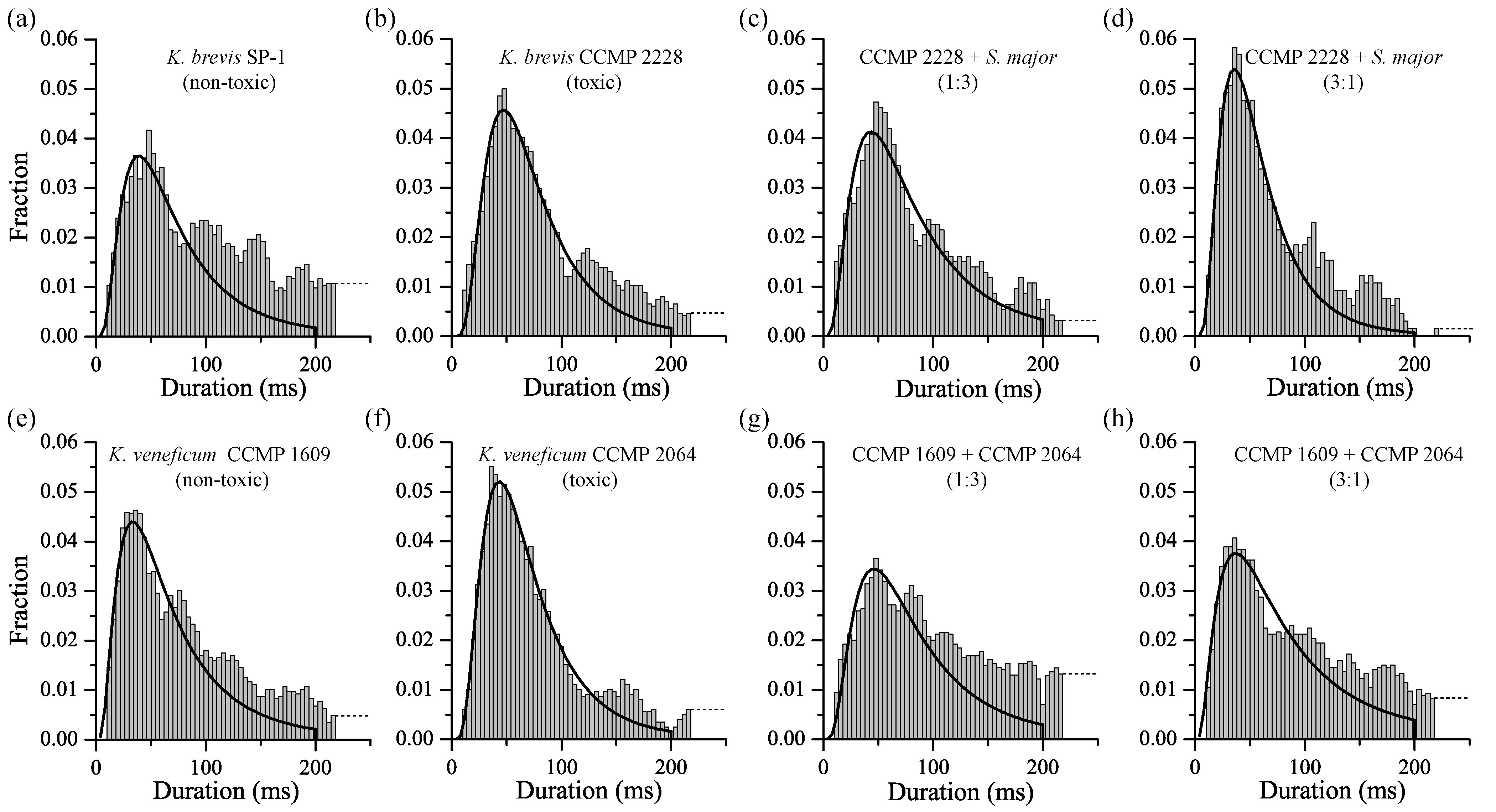


**Figure S3**. Sampling beating duration histograms for *A. tonsa* on mono-algal and mixed diets of *K. brevis* and *K. veneficum* focusing on the 0-200 ms range with 4 ms bins. The solid curve is a log-normal fit to the 0-100 ms range, which is terminated at 200 ms, and is used as a model for sampling duration.

**Table S8**. **Logarithmic fitting parameters for sampling beating duration histogram**

|  | no prey | *S. major* | *Karenia brevis* | | | | *Karlodinium veneficum* | | | |
| --- | --- | --- | --- | --- | --- | --- | --- | --- | --- | --- |
|  |  |  | SP-1 (non-toxic) | 2228 (toxic) | 2228+*S.major* | | 1609 (non-toxic) | 2064 (toxic) | 2064+1609 | |
|  |  |  |  |  | 1:3 | 3:1 |  |  | 1:3 | 3:1 |
| Geometric mean $\mu_{g}^{s}$ (ms) | 54 | 75 | 61 | 65 | 69 | 52 | 56 | 61 | 71 | 69 |
| Geometric std $\sigma_{g}^{s}$ | 1.88 | 2.19 | 1.95 | 1.75 | 1.98 | 1.80 | 2.07 | 1.78 | 1.94 | 2.22 |
| The peak location of fitting curve (ms) | 38 | 42 | 40 | 48 | 43 | 40 | 38 | 43 | 47 | 38 |
| The peak location of original histogram (ms) | 40 | 44 | 48 | 48 | 48 | 40 | 40 | 36 | 48 | 36 |
| rms fitting error in 0-100 ms (%) | 0.2 | 1 | 2.6 | 0.9 | 2.2 | 0.7 | 1.6 | 0.3 | 2.5 | 1.3 |
