# Supplementary material for: Algal Toxins Alter Copepod Feeding Behavior
Source: PLoS One. 2012 May 18;7(5):e36845. doi: 10.1371/journal.pone.0036845 (PMC3356345; doi:10.1371/journal.pone.0036845)
Supplement: Supporting Information S6 — Grazing beating duration histograms for A. tonsa on K. brevis and K. veneficum diets. (DOC) [file pone.0036845.s006.doc]

**Supporting Information S6: Grazing beating duration histograms for *A. tonsa* on *K. brevis* and *K. veneficum* diets**

Figure S4 shows the entire set of grazing beating duration histograms for *A. tonsa* on *K. brevis* and *K. veneficum* diets. As indicated in Table S9, log-normality is achieved for most of the diets with an rms fitting errors of 8% or less, except for the two mixed-algal diets involving the *K. brevis* 2228 (Figure S4c and d). In these two cases, the histograms deviate from the log-normal distribution significantly with rms fitting errors of 18% and 90%, respectively. The properties of the fitted curve for each diet case are listed in Table S9.


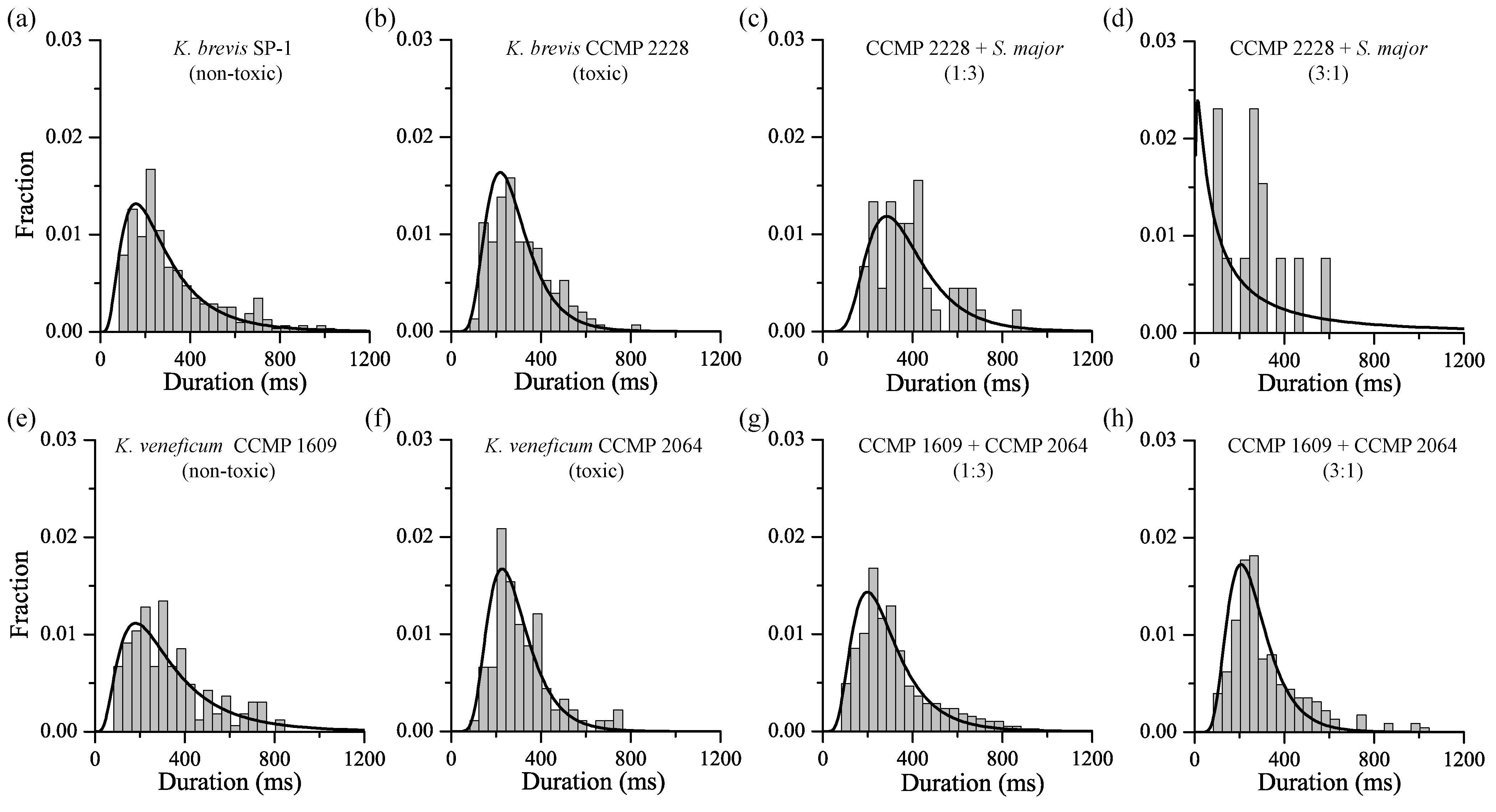


**Figure S4**. Grazing beating duration histograms for *A. tonsa* on mono-algal and mixed diets of *K. brevis* and *K. veneficum* with 40 ms bins. The solid curve is a log-normal least square fit to the histogram.

**Table S9**. **Summary of logarithmic fitting parameters for grazing beating duration histogram**

|  | no prey | *S. major* | *Karenia brevis* | | | | *Karlodinium veneficum* | | | |
| --- | --- | --- | --- | --- | --- | --- | --- | --- | --- | --- |
|  |  |  | SP-1 (non-toxic) | 2228 (toxic) | 2228+*S.major* | | 1609 (non-toxic) | 2064 (toxic) | 2064+1609 | |
|  |  |  |  |  | 1:3 | 3:1 |  |  | 1:3 | 3:1 |
| Geometric mean $\mu_{g}^{g}$ (ms) | N/A | 347 | 235 | 259 | 342 | 169 | 275 | 264 | 254 | 245 |
| Geometric std $\sigma_{g}^{g}$ | N/A | 1.66 | 1.88 | 1.51 | 1.54 | 4.97 | 1.91 | 1.48 | 1.64 | 1.51 |
| rms fitting error % | N/A | 4.2 | 5.8 | 7.3 | 17.6 | 89.9 | 8.3 | 8.3 | 4.0 | 6.8 |
