# Supplementary material for: Algal Toxins Alter Copepod Feeding Behavior
Source: PLoS One. 2012 May 18;7(5):e36845. doi: 10.1371/journal.pone.0036845 (PMC3356345; doi:10.1371/journal.pone.0036845)
Supplement: Supporting Information S7 — Sensitivity to mode separation criteria and procedure. (DOC) [file pone.0036845.s007.doc]

**Supporting Information S7: Sensitivity to mode separation criteria and procedure**

To demonstrate the robustness of trends shown in Figure 6a, we examine the effect of using a different mode separation criterion, consisting of a single duration cutoff, namely the beatings with duration below a threshold are considered as sampling while those above it are considered as grazing. Thresholds of 100, 150 and 200 ms have been tested for all the diet cases, and results are presented together with the data from the Figure 6a in Figure S5a. Evidently, the mode separations based on these alternative criteria do not change the trends of time allocation for sampling and grazing based on Figure 6a, but only shift all the symbols together towards higher sampling and lower grazing, or the other way around. For instance, the high sampling and low grazing fractions are still clear for the two toxic mono-algal diet cases, so do the distinct beating trends of *A. tonsa* on two types of mixed-algal diets. In addition, instead of using grazing beating fraction as the vertical axis, Figure S5b shows the total beating fraction *vs.* sampling beating fraction for all diet cases, and the trends are clearly consistent with the results shown in Figure 6a.


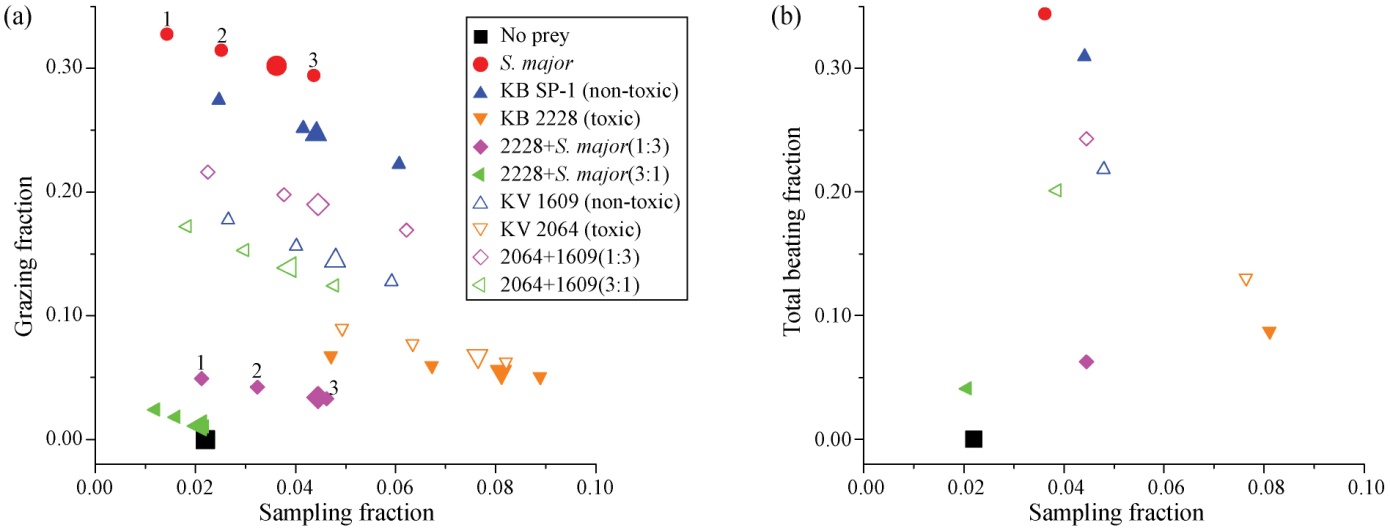


**Figure S5**. (a) The sampling and grazing fraction of *A. tonsa* using different mode separation criteria. The number on top of the symbol indicates that the data point is obtained using a single threshold duration, above which the beating is considered as grazing, of 100 ms ('1'), 150 ms ('2'), and 200 ms ('3'). The symbols with larger size are the data in Figure 6a obtained using the mode separation approach introduced in the main text. (b) The total beating fraction plotted against sampling fraction of *A. tonsa* on all diets using the same mode separation criterion as the one used for the data in Figure 6a.
