# Supplementary material for: Algal Toxins Alter Copepod Feeding Behavior
Source: PLoS One. 2012 May 18;7(5):e36845. doi: 10.1371/journal.pone.0036845 (PMC3356345; doi:10.1371/journal.pone.0036845)
Supplement: Supporting Information S8 — Food rejection and selective feeding behavior. (DOC) [file pone.0036845.s008.doc]

**Supporting Information S8: Food rejection and selective feeding behavior.**

Video S4 shows that a toxic *K. brevis*2228 cell becomes inadvertently entangled with its feeding appendages, the copepod tries to push the dinoflagellate away. To keep the dinoflagellate in focus, the holographic reconstructed images shown are compressed over a depth of 400 μm, i.e. they are an average of 20 images reconstructed in depth intervals of 20 μm.

Video S5 shows that *A. tonsa* directs its feeding current to entrain a *S. major* cell while ignoring a *K. brevis*2228 swimming right above its mouthpart. To keep both dinoflagellates in focus, the holographic reconstructed images shown are compressed over a depth of 400 μm, i.e. they are an average of 20 images reconstructed in depth intervals of 20 μm.
