# Supplementary material for: Algal Toxins Alter Copepod Feeding Behavior
Source: PLoS One. 2012 May 18;7(5):e36845. doi: 10.1371/journal.pone.0036845 (PMC3356345; doi:10.1371/journal.pone.0036845)
Supplement: Supporting Information S9 — Bootstrap analysis of sampling and grazing beating fraction. (DOC) [file pone.0036845.s009.doc]

**Supporting Information S9: Bootstrap analysis of sampling and grazing beating fraction**

Table S10 provides results of bootstrap analysis [1] to determine the statistical convergence of sampling and grazing fraction presented in Table 2. The numbers presented in brackets near each value show the 95% confidence interval based on bootstrap tests of the mean values.

**Table S10. Summary of sampling and grazing beating statistics**

|  | no prey | *S. major* | *Karenia brevis* | | | |
| --- | --- | --- | --- | --- | --- | --- |
|  |  |  | SP-1 (non-toxic) | 2228 (toxic) | 2228+*S.major* | |
|  |  |  |  |  | 1:3 | 3:1 |
| Sampling beating fraction | 0.022 [0.021, 0.023] | 0.039 [0.036, 0.043] | 0.052 [0.047, 0.058] | 0.053 [0.051, 0.056] | 0.036 [0.034, 0.038] | 0.024[0.022,0.027] |
| Grazing beating fraction | 0.000 | 0.281 [0.257, 0.304] | 0.209 [0.191, 0.226] | 0.082 [0.070, 0.091] | 0.042 [0.028, 0.047] | 0.010 [0.006,0.011] |
| Corr. sampling fraction | N/A | 0.036 [0.033, 0.040] | 0.044 [0.040, 0.049] | 0.081 [0.078, 0.087] | 0.045 [0.042, 0.048] | 0.021 [0.019, 0.023] |
| Corr. grazing fraction | N/A | 0.302 [0.276, 0.328] | 0.248 [0.226, 0.267] | 0.053 [0.046, 0.059] | 0.034 [0.024, 0.039] | 0.011 [0.007, 0.013] |

|  | *Karlodinium veneficum* | | | |
| --- | --- | --- | --- | --- |
|  | 1609 (non-toxic) | 2064 (toxic) | 2064+1609 | |
|  |  |  | 1:3 | 3:1 |
| Sampling beating fraction | 0.059 [0.056, 0.064] | 0.069 [0.065,0.075] | 0.049 [0.046, 0.054] | 0.049 [0.046, 0.053] |
| Grazing beating fraction | 0.118 [0.099, 0.133] | 0.073 [0.055,0.094] | 0.174 [0.156, 0.188] | 0.109 [0.095, 0.118] |
| Corr. sampling fraction | 0.048 [0.045, 0.052] | 0.076 [0.071, 0.083] | 0.044 [0.042, 0.049] | 0.038 [0.036, 0.041] |
| Corr. grazing fraction | 0.146 [0.123, 0.165] | 0.066 [0.049, 0.086] | 0.190 [0.171, 0.206] | 0.139 [0.121, 0.152] |

**References**

1. Davison AC, Hinkley DV, Canty AJ (1999) Bootstrap methods and their application: Cambridge University Press.
